# Behaviour and welfare of African lion (*Panthera leo*) cubs used in contact wildlife tourism

**DOI:** 10.1017/awf.2023.24

**Published:** 2023-03-27

**Authors:** Ann Wilson, Clive JC Phillips

**Affiliations:** 1 AW Applied Behavioural Ecology and Ecosystem Research Unit, Department of Agriculture and Environmental Sciences, University of South Africa, Florida, South Africa; 2 CJCP Estonian University of Life Sciences, Institute of Veterinary Medicine and Animal Sciences, Kreutzwalki 1, Tartu 51014, Estonia and Curtin University Sustainability Policy (CUSP) Institute, Curtin University, Perth, Western Australia 6845, Australia

**Keywords:** activity budgets, animal welfare, behaviour, lion cub interactions, South Africa, wildlife tourism

## Abstract

Lion (*Panthera leo*) cubs are used in wildlife interaction tourism but the effects on cub welfare are unknown. We assessed the behaviour of three cohorts of lion cubs, twelve animals in total, at three different interaction facilities, using continuous and scan-sampling methodologies for the entire duration of cub utilisation for human interactions. Cubs spent most time inactive (62%), particularly sleeping (38%), but also spent a substantial amount of time playing (13%) and being alert (12%). A generalised linear mixed model revealed that cub behaviour was similar in two facilities but different from cubs in the third. In these two similar facilities, as human interactions increased, the time spent resting, sleeping and playing with other cubs decreased, and alert behaviour, grooming of humans and flight responses increased. In the third facility, cubs had an abnormal activity budget, with high levels of inactivity (80%) accompanied by a lack of response to human interactions. We conclude that in some facilities normal cub behaviour cannot be achieved and may be compromised by a high frequency of human interactions, which therefore needs to be controlled to limit adverse effects on cub behaviour.

## Introduction

While education, research and conservation are common objectives of modern zoos, so too is entertainment, with visitors seeking to not only learn about and view the animals from a distance, but to also interact with them in close proximity (Fernandez *et al.* 2009). Interacting with wildlife has as a result become a part of many modern zoos and aquaria, with the public being willing to pay extra for the experience (Kreger & Mench 1995). Interacting with wildlife is not restricted to zoos but also exists within a commercial environment for recreational purposes (Moorhouse *et al.* 2017), such as swimming with dolphins (*Tursiops truncatus*), tiger-petting (*Panthera tigris)*, selfies with sloths (*Bradypus variegatus*) and elephant (*Elephas maximus*) riding, to name just a few. These practices are driven by tourist demand, many of whom find interacting with a wild animal enjoyable (Shani 2009).

Lion (*Panthera leo*) cub interaction encounters is a particularly popular international tourism activity (Wilson & Phillips 2021). While exact numbers are unknown, it has been estimated that up to 10,000 lions are used internationally, with 500,000 annual visitors (Moorhouse *et al.* 2017). Such tourism would include interacting with lions through petting and feeding, photographic opportunities such as selfies and walking with lions and visiting zoos and sanctuaries. There are approximately 7,000 lions in captivity in South Africa (Nowark 2015), owing to the captive breeding and commercial utilisation (in addition to the tourism uses referred to, this includes hunting and the farming of lion for the lion bone trade) of the species. Ethical debates in the media have brought the attention of the public to the use of lion cubs for interactions (TripAdvisor 2016; Africa Geographic 2020; Getaway 2020). Although the impact of visitors on cub behaviour and welfare has not been studied, we reported the output of a stakeholder workshop and conjoint analysis survey, which identified and ranked welfare issues faced by lion cubs within this tourism interaction industry (Wilson & Phillips 2021). While the key issue identified was a lack of governance of the industry, there are some other key issues relating to cub behaviour, specifically the cubs’ ability to choose their environment and escape from the interactions, keeper understanding of cub behaviour, and cubs’ sleep and social needs. This study, as a follow-up, attempts to answer what impact human interactions, and specifically the frequency of such interactions, have on cub behaviour and what that may mean for cub welfare.

Wild animals in their natural environments will typically exhibit avoidance behaviours when confronted by humans. However, when in captivity, wild animals exposed to frequent human presence may become tame, characterised by a decreased fear of humans (Hediger 1964). Over time, husbandry has the capacity to produce tamer individuals (McDougall *et al.* 2006), but this is highly dependent on management systems (Tennessen & Hudson 1981) which, in turn, has associated welfare implications (Dawkins 1980). Poor husbandry is most likely to generate stress in untamed individuals (McDougall *et al.* 2006). Animals involved in zoo interaction experiences are exposed to people in a different way to animals in other contexts, such as farms and laboratories, which have small groups of familiar people taking care of them (Price 1984). Animals used in interactions are additionally exposed to large numbers of unfamiliar people, who behave in an unpredictable manner and respond to them uniquely (Hosey & Melfi 2015).

Proximity to humans and extensive social interactions with them induce stress responses in many species (Fernandez *et al.* 2009). These responses not only differ between species but also between individuals within a species (Hosey 2008) and such individual differences should be considered in the management of these animals (Wielebnowski 1999). It is even recommended that these individual responses should give rise to tailored welfare treatments for individuals that respond differently from the average (Richter & Hintze 2019). Adaptive behavioural responses of animals can relate directly to management strategies, with responses being shown by the animals to either real (fear) and/or perceived (anxiety) threats (Boissy 1995). Fear and anxiety in animals are undesirable emotional states which reduce welfare (Boissy 1995).

Visitors to zoos have variable effects on the animals’ welfare, known as the ‘visitor-effect’, with both behavioural and physiological components (Davey 2007). The impact is not always negative such as is seen with avoidance and aggressive behaviours, or even neutral as in habituated animals, but may even be positive, such as in attention-seeking behaviours (Sherwen & Hemsworth 2019). However, this visitor-effect is not easy to evaluate (Davey 2007) and, while visitor behaviour and animal behaviour are associated, it is difficult to show causality (Margulis *et al.* 2003). This may be as a result of animals causing a behavioural response in humans and *vice versa*, known as ‘visitor attraction’ (Davey 2006). This bi-directional relationship will usually have a primary direction and is taxon-specific (Margulis *et al.* 2003). No studies of this visitor effect on the African lion were able to be sourced but one was conducted on the Asiatic lion (Suárez *et al.* 2017). This indicated that there was no correlation between visitor density and Asiatic lion activity and concludes that the animals were affected by the mere presence of humans irrespective of their number, with solutions needed to address such negative effects.

To measure management effects on welfare, the cumulative effects of husbandry choices on the cub’s behaviour must be assessed. The principle of additivity of multiple concurrent stressors has been demonstrated in the livestock production industry (e.g. Hyun *et al.* 1998). At lion cub interaction facilities, the effect of keeper relationships, interaction numbers and frequency, interactor behaviour, the weaning age of the cubs, starting age of interactions and effects of training styles all have the potential to affect the behaviour of the cubs in a cumulative manner.

Animal keepers and their manner of handling farm animals has been linked to productivity, with a fear of humans, derived from rough and inappropriate handling, resulting in effects such as chronic stress, reduced reproduction rates in pigs (*Sus scrofa*), poultry and foxes (*Vulpes vulpes*) and decreased milk production in dairy cows (*Bos taurus*) (Carlstead 2009; Zulkifli 2013). The quality of relationships between zoo animals and their keepers can affect reproductive success, with responses to positive keeper time and contact (Mellen 1991). Positive relationships can improve social behaviours amongst chimpanzees (*Pan troglodytes*) and decrease abnormal behaviour (Baker 1994) as can positive reinforcement training (Pomerantz & Terkel 2009). Behavioural responses of zoo animals towards the interacting public are also dependent on the quality of interactions (Hosey & Melfi 2015). Learned helplessness is a potential negative response which can be expected in wildlife interactions and has been evident in wombats (*Lasiorhinus latifrons*) used in human interaction encounters (Hogan *et al.* 2011). Crowd size, visitor frequency and proximity to the zoo animal being interacted with all affect behavioural responses (Fernandez *et al.* 2009). Behaviours are influenced by the personalities of the individual animals (Gosling *et al.* 2003), modified by their temperament and past experiences. Animals weaned early and provided with reconstituted milk are likely to be more motivated to interact with humans than those naturally suckled (Jago *et al.* 1999), as are animals which are handled from an early age (Markowitz *et al.* 1988). A young, still developing animal is especially sensitive to human stimulation and this impact, either positive or negative, can have a long-lasting impact on the animal, including its genetic potential (Zulkifli 2013). It has even been suggested that repetitive negative interactions may prevent the development of future positive interactions (Hosey & Melfi 2015), and this is concerning for an animal facing a lifetime in captivity. Therefore, assessing the welfare of lion cubs used in interaction facilities requires investigation into the effects of management on their behaviour, with the aim of inferring their resultant emotional states.

The main objective of our study was to investigate the behaviour of lion cubs used in wildlife tourism interaction facilities and then to specifically examine behavioural responses to human interaction frequency, with the aim of detecting and describing any possible problem behaviours.

## Material and methods

Three South African lion cub tourism facilities offering very different interaction experiences were used in the study. Names and locations have been withheld to preserve their anonymity. Ethical clearance was granted for the study by the University of South Africa’s College of Agriculture and Environmental Sciences, Animal Research Ethics Committee (reference number: 2017/CAES/053).

Continuous sampling was used across the three facilities, irrespective of their management styles. We recorded durations and time budgets of all behaviours, which were mutually exclusive (Hartmann 1982). Each sampling day had two recording periods, 0900–1200 and 1300–1600h. The behaviour of each cub was recorded continuously for a 10-min period within each hour, totalling 60 min per cub per day. Study sites were visited three or four times per month for the period that the cubs were used for interactions, with data being collected during both weekdays (when the facilities received fewer interactors), and on weekends and public holidays (when there were increased numbers of interactors), in order to attempt to determine the relationships between interaction numbers and behaviours displayed by the cubs. Human interaction frequency relates to the number of human interactors which entered the lion cubs’ enclosure during the two recording periods within a given sampling day. The behaviour of all of the study cubs within each facility were also scan sampled at 10-min intervals during the same two recording periods as the continuous sampling. This resulted in 36 scans per cub per recording day. The scan sampling was however only used to add context and description to rare behaviours observed.

Cubs were sampled in a random order (determined before starting the day’s recordings and this order was kept for each subsequent recording), and to minimise observer influence, the observer arrived 30 min prior to data collection, to allow the cubs to habituate to her presence. The observer watched the behaviour from inside the enclosure, with a hide deemed unnecessary because the cubs were well habituated to the presence of humans on account of their daily contact with humans. The researcher never interacted with the cubs and moved around within the enclosure to ensure that she could see all areas accessible to the cubs. This was especially important during interaction sessions with several people in the enclosure at the same time. The cubs never approached the researcher, and this was considered a necessity for accurate data collection. Inter-rater differences were eliminated by only using one observer (Hartmann 1982). Trial runs during the construction and testing of the ethogram were used to train the observer. Cubs could be easily identified, making use of unique distinguishing characteristics such as colour, size, sex, hair patterns etc.

Data collection commenced as new cubs became available and therefore did not occur for exactly the same time-period for the three facilities. The number of recording days was determined by the length of time cubs were used in the activities (Table 1). Facility A comprised three female sibling cubs that were recorded altogether on ten days (four weekdays, five weekend days and one public holiday) over a 13-week period during April to June 2017. Facility B had four cubs, three siblings (one female and two males) and a single cub (male) from another litter, that were recorded on 15 days (ten weekdays and five weekend days) over a 21-week period during May to October 2017. Facility C had five cubs, two siblings (one male and one female) and three single cubs all from other litters (two males and one female) that were recorded on 12 days (eight weekdays, three weekend days and one public holiday) in a 21-week period between August 2017 and March 2018. Cubs were all hand-reared, and bottle-fed within a few days of birth.

**Table 1.** The three lion cub facilities included in the study and the differences in the management of the lion cub interactions

Management action	Facility A	Facility B	Facility C
**Cubs**			
Cub starting age of interaction	34 days	34–38 days	57–103 days
Cub ending age of interaction	100 days	189–193 days	182–271 days
**Interactions**			
Interaction periods with the public	Predominantly weekends and public holidays; minimal midweek activity, anytime between 0800–1700h	Two set interaction sessions every day, at 0900 and 1500h	Predominantly continuous, every day of the week, from 0800–2000h
Number of interactors within a recording day	Mean: 24; range: 0–65	Mean: 31; range: 9–71	Mean: 102; range: 0–298
Extent of interaction	Unrestricted	Head and back petting encouraged but other interactions allowed if not aversive to cub	Head and back petting only, but occasionally deviated from when not observed by handler
Discipline	None by handler; occasionally by interactor when not observed by handler	Tap on the nose by handlers and communicated as acceptable for the interactor to use if required	Control seldom required by handlers due to inactive nature of cubs
**Staff**			
Rearing and feeding of cubs	Keeper	Volunteers	Keeper
Cleaning enclosure	Non-interacting staff	Non-interacting staff	Non-interacting staff
Managing interactions	Keeper	Handlers	Handlers

An ethogram (Table 2) was used to record cubs’ common behaviours, constructed using the available lion ethology literature (Schaller 1972; Bertram 1975; Estes 2012). A pilot study allowed for the testing of the ethogram and the adding of any new observed behaviours.

**Table 2.** Behaviour categories and detailed descriptors used to record duration of daily behaviour of the cubs

Behaviour	Descriptor
**Play**	A voluntary action including running, quick turns, rolling, climbing and wrestling (Estes 2012; Bertram 1975)
Self-play	Play by self, not involving any other living animal and or human
Conspecific play	Play with other lion cubs
Human play	Play with humans
**Inactivity**	Limited or no movement
Sleeping	Eyes closed, lying laterally or sternally recumbent
Resting	Eyes open but non-responsive to surroundings
**Locomotion**	Moving from one place to another, not part of play, grooming, aggression, submissive or excited behaviours
Flight	Alarmed reaction and hasty retreat from a perceived or real threat, to a new location
Movement	Purposefully walking or running to a new location
**Grooming**	Cleaning or maintenance of the body, involving head rubbing and social licking (Schaller 1972)
Autogrooming	Grooming behaviours only directed to self
Allogrooming	Grooming behaviours directed at other lion cubs
Human grooming	Grooming behaviours directed to a human
**Aggression**	Threatening or contact behaviour, which may or may not be harmful, includes growling, lunging, swiping, biting or a combination of these
Conspecific aggression	Aggressive behaviour directed at another lion cub
Human aggression	Aggressive behaviour directed at a human
**Abnormal behaviours**	Behaviour which deviates from normal and appears to have no obvious goal or function
Stereotypy	A repetitive behaviour such as pacing back and forth, usually along an enclosure edge, may include vocalisation
Non-nutritive sucking	The act of sucking another cubs’ ear or genitalia
Shaking and trembling	An involuntary behaviour resulting from a perceived threat indicating fear
Fence-biting	Biting on the fence of the enclosure but not in the same manner as during teething
**Attentive**	Reacting to its surroundings
Alert	Cub is watchful of surroundings, although not moving. The eyes are focused on environmental stimuli and ears move responsively to noises
Investigatory	Cub actively explores its environment by using its senses
Excited	Cub moves excitedly, sometimes vocalising and mostly in a small area around an object or person
**Miscellaneous**	
Mock mating	The imitating act of mating, not gender-specific
Flehmen	Wrinkles the nose and curls the top lip in response to a smell, usually other cubs’ urine
Elimination	The act of defaecating and urinating and includes the act of being stimulated when a young cub by the caretakers to encourage bowel movement
Ingestion	The active consumption of food and liquid; includes drinking of water, bottle-fed milk, any solid food such as cat pellets, chicken and meat and includes chewing on a carcase

### Statistical analysis

A Generalised Linear Mixed Model with a negative binomial distribution and a log-link function was used to ascertain any relationship between the variables tested. Facility and Behaviour, as well as the interaction between these two, were included as the main effects in the model as differences between the facilities were likely to influence the behaviour expressed by the cubs. The individual animal, its sex and the observation day were included as random factors (they were not the focus of the study), and cub age and total number of people that encountered the cub’s enclosure within each recording day were included as continuous predictors within the model (as likely to effect behaviour, with number of people being the focus of the study). Repeated measures of each animal were accounted for statistically by nesting observation day within individual animal effects. The counts of each behaviour category were used as the response variable in the model. The glmmTMB package in R (Brooks *et al.* 2017) was used to perform the analyses. Model structures were compared using the Wald χ^2^ test, through analysis of deviance type 3 testing, using the ANOVA function in the ‘car’ package (Fox & Weisberg 2019), to investigate patterns of predictor significance.

For all significant predictors, pair-wise comparisons were performed using the Wilcoxon rank-sum tests, to identify significant differences between factor states. All tests were two-tailed and considered significant at *P* < 0.05. *P*-values for the outcomes of the tests were adjusted for multiple test comparisons using the p.adjust() function in R (R Core Team 2013) and the Benjamini-Hochberg correction method (Benjamini & Hochberg 1995). The continuous predictor, cub age, was associated with a significant effect across facilities and so each broad ethological grouping was correlated (Pearson’s correlation coefficient) and individually tested for significance.

A percentage activity budget (indicating the mean, SD and SEM) was constructed for all behaviours derived from the three facilities used in the study. All behaviour frequencies were tested against human interaction frequency, to ascertain strengths of correlations and significance within Facilities.

## Results

Each facility was observed to manage their cubs and the interaction experience in a unique way. They differed in the number and type of staff responsible for the cubs’ care, the ages of cubs used, their duration and frequency of interactions, the manner of the interaction allowed, and the disciplining of the cubs by staff (Table 1).

An activity budget of the lion cubs studied at the three interaction facilities (Table 3) shows large variation in behaviour, with big SD values, but the low SEM values give confidence in the data (Barde & Barde 2012).

**Table 3.** Activity budget as % of total time (sample mean; SD; SEM) of lion cubs (n = 12) used in three wildlife tourism interaction facilities

Behaviour	Sample mean (% of total time)	SD (% of total time)	SEM (% of total time)
Inactive	62.14	19.75	1.71
Sleeping	38.20	18.71	1.61
Resting	23.94	12.12	1.04
Play	13.35	11.22	0.97
Self-play	6.19	7.408	0.552
Conspecific play	5.54	6.417	0.552
Human play	1.62	3.049	0.262
Attentive	13.12	8.41	0.73
Alert	12.17	7.81	0.67
Investigatory	0.36	1.027	0.088
Excitement	0.59	2.344	0.202
Locomotion	6.21	4.346	0.377
Flight	0.24	0.551	0.047
Movement	5.97	4.091	0.352
Grooming	1.42	1.831	0.159
Autogrooming	1.06	1.690	0.145
Allogrooming	0.28	0.704	0.061
Human grooming	0.08	0.401	0.034
Abnormal	0.82	3.460	0.300
Stereotypy	0.55	3.329	0.286
Non-nutritive sucking	0.22	0.828	0.071
Shaking and trembling	0.04	0.488	0.042
Fence-biting	0.01	0.141	0.012
Aggression	0.41	0.872	0.076
Conspecific aggression	0.26	0.660	0.057
Human aggression	0.15	0.524	0.045
Miscellaneous	2.52	5.085	0.441
Mock mating	0.00	0.017	0.002
Flehmen	0.02	0.207	0.018
Elimination	0.13	0.301	0.026
Ingestion*	2.37	4.966	0.427

* Ingestion not a true representation as may have occurred outside the recording periods

**Figure 1.**
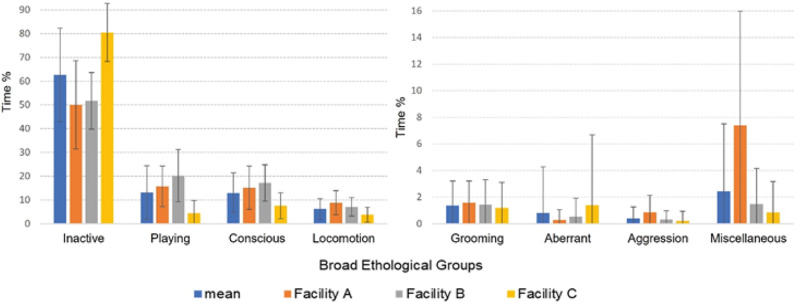
Sample mean (± SD) percentages of time spent in behaviour categories by the lion cubs across the three interaction facilities.

Graphical depiction of the mean values for behaviour of the cubs at the three facilities (Figure 1) suggests that cubs in Facilities A and B were similar to each other in their behaviour, and Facility C differed from these two for certain behaviours. This visual interpretation is supported by the statistical analysis of the interaction between ‘Facility and Behaviour’, which was a significant predictor of the Generalised linear mixed model outcomes (Wald χ^2^_14_ = 100.78; *P* < 0.001).

Wilcoxon rank sum tests applied to each pair of facilities revealed that Facilities A and B were significantly different to Facility C (*P* < 0.005), but not significantly different to each other, for behaviour categories inactive, play, attentive, locomotion and aggression. Grooming and abnormal behaviour categories were not significantly different between Facilities (Table 4). As such, Facility A and B are statistically grouped together as Facility pair AB when determining a response to human interaction frequency, whilst Facility C remains independent in its response.

**Table 4.** Wilcoxon rank sum test statistics for each pair of Facilities (AB, AC and BC) to determine significant differences across behaviour categories

Behaviour	Facility pairs		
Categories	AB	AC	BC
Inactive	W = 676, adj. *P* = 0.599	W = 1273, adj. *P* < 0.005	W = 2667, adj. *P* < 0.005
Play	W = 580, adj. *P* = 0.137	W = 187, adj. *P* < 0.005	W = 231.5, adj. *P* < 0.005
Attentive	W = 619, adj. *P* = 0.273	W = 348.5, adj. *P* < 0.005	W = 451.5, adj. *P* < 0.005
Locomotion	W = 876, adj. *P* = 0.142	W = 256, adj. *P* < 0.005	W = 689.5, adj. *P* < 0.005
Grooming	W = 846, adj. *P* = 0.293	W = 493, adj. *P* = 0.089	W =1240.5, adj. *P* = 0.293
Abnormal	W = 687, adj. *P* = 0.944	W =706, adj. *P* = 0.962	W =1343.5, adj. *P* = 0.944
Aggression	W = 941.5, adj. *P* = 0.031	W = 363.5, adj. *P* < 0.005	W = 962.5, adj. *P* < 0.005

### Human interaction frequency

**Figure 2.**
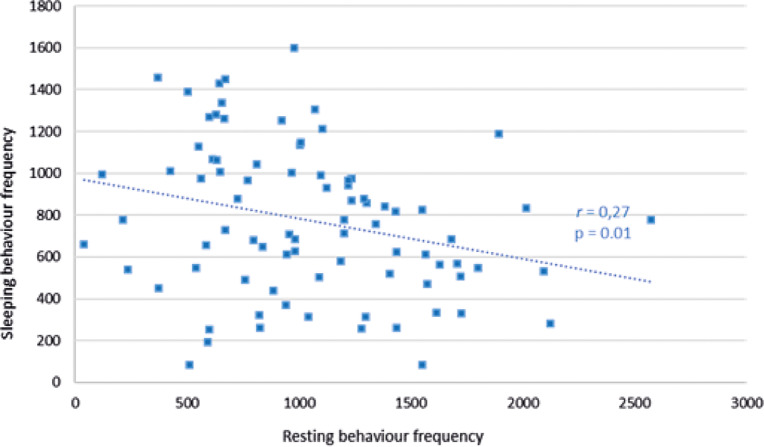
Relationship between sleeping behaviour frequency of cubs and the resting behaviour frequency of cubs within a recording day at Facilities pair AB.

**Figure 3.**
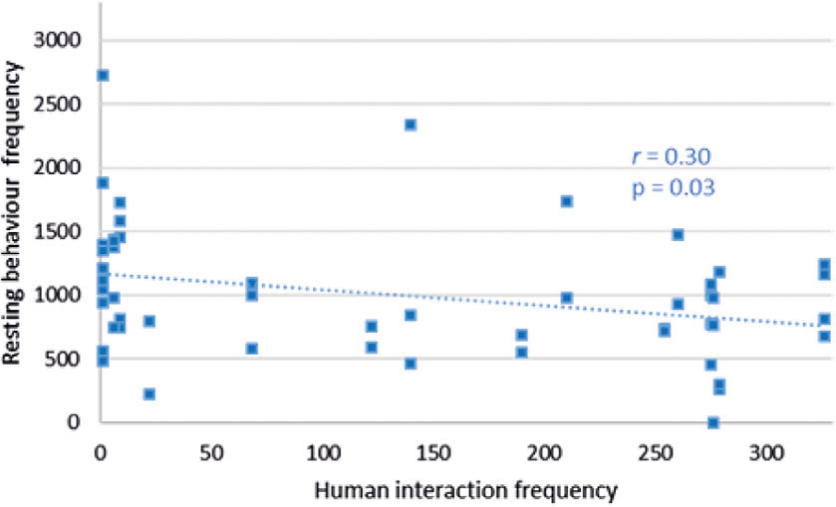
Relationship between resting behaviour frequency of cubs and human interaction frequency within a recording day at Facility C.

In Facilities pair AB there was a trend (*r*_79_ = –0.19; *P* = 0.08) for sleeping behaviour, but not resting behaviour (*r*_79_ = +0.16; *P* = 0.15), to decline with increased human interaction frequency. Sleeping times were significantly negatively associated with resting times at Facilities pair AB (*r*_79_ = –0.27; *P =* 0.01) (Figure 2). Sleeping behaviour at Facility C did not correlate with human interaction frequency (*r*_50_ = +0.03; *P =* 0.86), but a decrease in resting behaviour was significantly negatively associated with an increase in human interaction frequency (*r*_50_ = –0.03; *P =* 0.03) (Figure 3).

**Figure 4.**
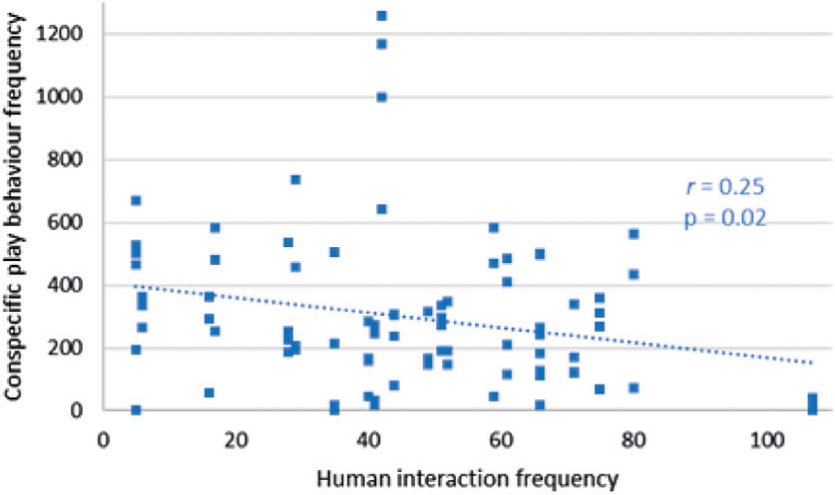
Relationship between conspecific play behaviour frequency of cubs and human interaction frequency within a recording day at Facilities pair AB.

Conspecific play behaviour decreased as human interaction numbers increased at Facilities pair AB (*r*_79_ = –0.25; *P =* 0.02) (Figure 4). Self-play behaviour frequency at this facility did not correlate with human interaction number (*r*_79_ = +0.14; *P =* 0.21) and nor did that of human play (*r*_79_ = +0.09; *P =* 0.44). Conspecific play at Facility C had no significant relationship with human interaction number (*r*_50_ = +0.22; *P =* 0.11), and neither did self-play (*r*_50_ = +0.22; *P =* 0.71). Play with humans at Facility C correlated positively with an increase in human interaction number (*r*_50_ = +0.27; *P =* 0.05).

**Figure 5.**
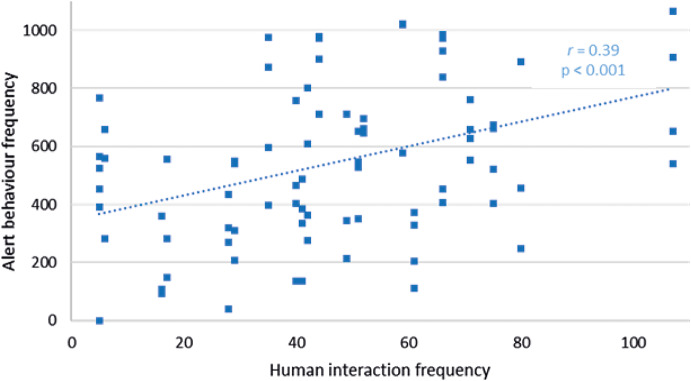
Relationship between alert behaviour frequency of cubs and human interaction frequency within a recording day at Facilities pair AB.

Of all the Attentive behaviours, only alert behaviour frequency had a significant relationship with human interaction frequency at Facilities pair AB (*r*_79_ = +0.39; *P <* 0.001), increasing at higher interaction frequencies (Figure 5). Investigatory behaviour (*r*_79_ = +0.01; *P =* 0.94) and excitement (*r*_79_ = +0.11; *P =* 0.31) were not significantly related to human interaction frequency in Facilities pair AB. Cub behaviour in Facility C was not significantly related to human interaction frequency: alert (*r*_50_ = +0.09; *P =* 0.52), investigatory (*r*_50_ = +0.16; *P =* 0.31), excitement behaviours (*r*_50_ = –0.14; *P =* 0.25).

Human grooming by cubs was positively correlated with increased human interaction frequency (*r*_79_ = +0.26; *P =* 0.02) in Facilities pair AB. Neither autogrooming (*r*_79_ = –0.13; *P =* 0.23) nor allogrooming (*r*_79_ = –0.07; *P =* 0.54) were correlated with human interaction frequency. Cubs in Facility C showed no associations between autogrooming (*r*_50_ = +0.03; *P =* 0.83), allogrooming (*r*_50_ = +0.10; *P =* 0.47) or human grooming (*r*_50_ = +0.02; *P =* 0.88) with human interaction frequency.

**Figure 6.**
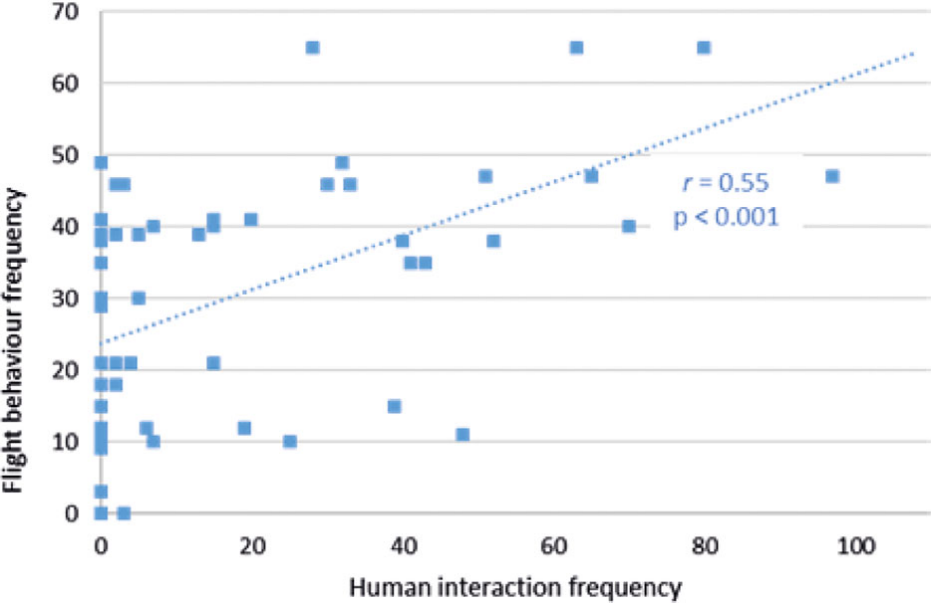
Relationship between flight behaviour frequency and human interaction frequency within a recording day at Facilities pair AB.

Increased human interaction frequency did not illicit an increase in either human-directed aggression (*r*_79_ = +0.09; *P =* 0.37) or conspecific aggression (*r*_79_ = +0.01; *P =* 0.90) in Facilities pair AB, or in Facility C (human-directed aggression [*r*_50_ = +0.14; *P =* 0.31], conspecific-directed aggression [*r*_50_ = +0.04; *P =* 0.80]). However, in Facilities pair AB (but not in Facility C [*r*_50_ = +0.02; *P =* 0.90]), flight behaviour frequency correlated positively with human interaction frequency (*r*_79_ = +0.55; *P <* 0.001) (Figure 6). This flight behaviour frequency in Facilities pair AB was correlated with human-directed aggression frequency in Facilities pair AB (*r*_79_ = +0.35; *P =* 0.001).

### Rare behaviours

Certain behaviours were too rarely recorded for statistical analysis and are only described. Pacing behaviour was determined as being different from the general movement behaviour category because it did not meet the requirements of purposefully moving from one location to an intended new one within the enclosure, as a result of its repeated pattern. It was also not included within the Attentive behaviour category due to the absence of any alert behaviour, investigatory or excited behaviour. It appeared to be unique to each facility in terms of causation and occurrence. Observations recorded during the scan-sampling method, and which did not form part of the data used in the General linear mixed model, are also included to add possible context to these rare behaviours. Facility A had only one incident of pacing, while pacing behaviour in Facility B only occurred when the cubs were confined to a travel crate (n = 2). One female cub at Facility B paced after being prevented by volunteers from performing non-nutritive sucking; she also paced during the time that a fire break was being burnt around the cubs’ enclosure. Cubs in Facility C exhibited the most pacing observations (n = 13) and for considerably longer periods of time (range 30–1,316 s). Most pacing behaviours were observed when cubs were separated from each other into an adjoining enclosure with a visual barrier between (n = 6). There was also deliberate pacing along a fence line (n = 2) with another pacing (n = 1) being displayed whilst a cheetah (*Acinonyx jubatus*) was hissing in a neighbouring new enclosure. In another pacing incident, loud noises made by trucks and machinery, outside of the enclosure coincided with pacing action (n = 1). Finally, pacing was also seen in a cub (n = 1), who recognised the keeper’s voice outside that of the enclosure.

Non-nutritive sucking may also be an indicator of a potential problem behaviour. All three female cubs at Facility A exhibited this behaviour and it was restricted to the sucking of ears. This sucking was observed six times (range: 4–120 s) and was no longer observed in these cubs after 66 days of age. Cubs in Facility B accounted for most (n = 15) non-nutritive sucking, consisting of both ear (33%) and genitalia (67%) sucking (range: 2–265 s). This behaviour was actively stopped by management and volunteers if seen, suggesting that more and longer durations of sucking could have been observed if the cubs had been allowed. Non-nutritive sucking of ears was last observed in a cub at this Facility at 133 days of age, and at 168 days of age for genitalia sucking. Facility C only had one recorded attempt to ear suckle, but after 2 s the 108 day old cub spat out the ear.

Lastly, two rarely observed problem behaviours were observed. A female cub at Facility B trembled for 204 s when she was 91 days of age. This was during the period described earlier, in which she paced due to a fire break being made around the enclosure she was in. This behaviour was not observed again. Another cub in Facility C was observed biting the fence for 59 s at 85 days of age, and again at 197 days of age (this observation lacks duration as it was recorded during a scan-sampling session).

## Discussion

Scientific literature on wild lion cub activity budgets is lacking, with behavioural studies of lions in the wild focusing almost exclusively on the adult lions within a pride. However, recognising that many behaviours are stimulus-driven (Stolba & Wood-Gush 1984), it is clear that wild lion cubs raised within a pride will experience vastly different internal and external stimuli to those raised in captivity, devoid of adult lion presence, but confronted by frequent interaction with humans. Consequently, resulting activity budgets from two very different stimuli bases would reflect equally different behavioural repertoires and frequencies. To compare activity budgets in this situation might therefore not serve to determine their state of welfare. The non-performance of a behaviour seen in the wild does not necessarily imply that welfare is compromised in the captive individual (Veasey *et al.* 1996) and it can be conversely deduced that new behaviours not being observed in wild conspecifics may not imply welfare compromise. Veasey *et al.* (1996) cautions that other techniques should be used in conjunction with wild behaviour comparisons in assessing welfare.

The extant scientific literature provides little information on inactive behaviour of lion cubs. The cubs within our study spent on average 62% of their time inactive, with the majority of this time spent sleeping. However, given that the behaviours were only recorded during the day, our data collection is not necessarily entirely representative. There is no consensus on the activity budgets of wild adult lions, even though it has been the subject of extensive research. Wild lions are easily identified from their distinguishing characteristics, such as markings that come with age, but cubs are more difficult to identify individually in the wild (Bertram 1975), making behavioural recording difficult for cubs, in the absence of invasive marking techniques. Hanby *et al.* (1995) reported that lions in Tanzania spent up to 80% of their time inactive, sleeping, lying down and sitting, mostly during the daytime. Hayward and Hayward (2006), however, found that lions in Addo National Park, South Africa, were active for 41% of each 24-h day, with one lioness active for as much as 54% of the time. They suggest that it is a popular misconception that lions sleep for 22 h per day. A study in Zimbabwe of self-sufficient (able to hunt for themselves) captive-born adults with their wild-born, sub-adult offspring, which were being prepared for release into the wild from a 403-acre fenced managed reserve, found that resting behaviour accounted for 61% of their time (range 52–69%). However, the activity budgets of these lions were probably influenced by research activities, such as playback of territorial vocalisation calls made by other lions (Dunston *et al.* 2016).

A study on lion cubs which collected data at 5-min intervals over three time-periods of 1 h each, during daylight hours, exists for comparison. Captive lion cubs in a sanctuary in Zimbabwe (Ncube & Ndagurwa 2010), mother-raised with no environmental enrichment, spent 68% of their time resting, i.e. much more than the cubs in our study. Within the same study, two separate orphaned lion cub groups not exposed to human interactions were provided with extensive environmental enrichment, which resulted in resting percentages of 37 and 40%, respectively. When comparing the inactive behaviour of the lion cubs in this study with other available literature, it is clear that even this relatively well understood and common behavioural category within ethological studies is unable to be compared due to limited relevant literature and/or an inconsistency in methodology. Dunston *et al.* (2017) conducted the first study to compare activity budgets between captive-origin and wild prides under the same methodology and determined that wild cubs rested less (on average 42%) than their captive counterparts (an average of 52%). Even under such a study of matching methodology it should be noted that the captive lions were observed more as a group than their wild counterparts and that this would affect interaction-type behaviours recorded. It was also determined that natural environmental conditions, such as closed riverbeds compared to grassland-type habitats, have effects on wild cub behaviour. As such, the activity budget of the lion cubs used in this study contributes new information about their behaviour under captive human interaction-specific environmental conditions. It must be reiterated that the focus of this study was to compare the behaviour of the cubs with themselves and each other under different human interaction frequencies. Environmental conditions such as weather, time of day and possible changes to their environments and even differences between their environments may also have influenced their behaviours, but these factors were not included as additional random effects.

The cubs at Facilities pair AB decreased sleep as human interaction frequency increased, correlating with their resting behaviour frequency increase. Wechsler (1995) explains how an animal’s behaviour has goal-related function and even coping behaviours are adapted to achieve these goals. So, even though Facilities pair AB cubs were able to maintain a level of inactivity, it is not a coping response, given that sleep and rest serve different biological functions. At Facility C, the leading behaviour in terms of time budget allocation was Inactivity at 80%, even accounting for as much as 100% of one cub’s sample budget day. A wide range of behaviours is required in order to alleviate suffering, with behavioural deprivation being classified as a central problem in animal welfare (Dawkins 1988), and inappropriate husbandry preventing animals from performing behaviours more typical of their species. Excessive sleep is identified as an avoidance strategy within the Stress Response Scale for humans, to avoid a stressful situation and its implications (Weiss *et al.* 1984). Bixler *et al.* (2005) determined depression to be the most significant risk factor associated with excessive sleep in people and that the association was stronger in the young. The Wistar-Kyoto (WKY) rat (*Rattus norvegicus*) is a genetic model in which excessive sleep and even narcolepsy have been associated with depression (Allard *et al.* 2004). MacLellan *et al.* (2021) reviewed evidence from several animal models of depression, as well as from animals experiencing poor welfare in captive conditions, and found that they exhibited depressive-like states, with low activity and unresponsiveness being one such state. Problem behaviours, such as excessive sleep, are reflective of poor mental states and poor welfare (Gonyou 1994). Inactivity can also be a biological indicator of boredom in animals (Burn 2017), which implies an awareness of self as the animal misses an opportunity to perform alternate behaviours (Wemelsfelder 1985). The corresponding negative emotion therefore associated with this behavioural problem is depression which, were it to continue, would result in suffering (Dawkins 1988). It would need to be ascertained whether the behavioural deprivation effect expressed by the cubs in Facility C is reversed once interactions are ceased or whether there is a long-lasting effect. Should it continue, then the malfunction-induced behaviour (Mason 2006) could morph into a stereotypical behaviour, with sleep acting as the continuous repetitive behaviour.

Play behaviour occurs once all primary needs have been met, similar to exploratory behaviour, indicating a positive internal state (Held & Špinka 2011). It encourages learning through behaviours adapted from usual contexts (Smith 1982), it strengthens social attachments (Bekoff 1977) and in correct contexts and quantities, reflects a positive emotional state (Barnett 1958). Play is a plausible indicator of welfare at a population level rather than at an individual level (Richter *et al.* 2016), as it has been known to increase when conditions are stressful, such as in the absence of parental care or after a period of deprivation (Held & Špinka 2011). Play therefore needs to be recorded in all of its various forms, such as social and solitary play, as well as frequencies of play and duration, if it is to be used to distinguish between different welfare states (Ahloy-Dallaire *et al.* 2018). Our cubs spent on average 13% of their time playing, evenly divided between conspecific play and self-play, with much less play with humans. Cubs in Facilities A and B decreased their conspecific play frequency as human interaction numbers increased. Human interactions may have prevented opportunities for conspecific play, yet human play did not correspondingly increase. Self-play at Facilities pair AB was not affected by human interaction increase, suggesting that human interaction numbers typically experienced at Facilities pair AB did not prevent learning within a solitary context, nor did they increase, as is often the case during stressful situations. Facility C cubs exhibited play behaviour at a frequency of less than one-third of those at Facilities pair AB. While their self and conspecific play were not affected by human interaction increase, their play with humans increased. It is possible that any increase of play with humans at Facility C may also be indicative of a stressful situation (Held & Špinka 2011), which would support the high levels of Inactivity being exhibited as a problem behaviour.

The behaviours within the Attentive behaviour category are biologically different but were grouped together, based on their focused state. Alert behaviour was the most common form of attentive behaviour expressed by the cubs. According to Wemelsfelder (1991), an animal should be able to be attentive when required to react to unexpected events and even concentrate on goal-oriented tasks. An inability to respond appropriately to environmental stimuli has been linked with a poor variability of behaviours, with animals in impoverished environments becoming habitually inattentive. Wemelsfelder (1989) suggests that this inattentiveness leads to an inability to adapt to an environment and can be a precursor to stereotypic behaviours (Wiepkema 1987). Facility C’s cubs were more than 50% less Alert than Facilities pair AB’s cubs. Ascertaining whether this was an effect of low behaviour variability, or simply a result of less time remaining after a large proportion had been assigned to inactive behaviour, is not as important as the resulting lack of alert behaviour, which is associated with poor welfare. The Investigatory behaviour shown by the cubs occurred very infrequently and with high variability. Its presence in an activity budget suggests positive welfare, given that the benefits of such behaviour outweigh any costs and risks to the individual (Renner 1990). The lack of the behaviour might constitute a lack of behavioural diversity. Excitement behaviour is a positive anticipatory and reward-seeking behaviour; a response to a physiological need associated with high-arousal positive states (Mendl *et al.* 2010). When there is a high expectation of positive events, emotions akin to optimism are experienced (Mendl *et al.* 2010). The presence of excitement behaviour within budgets of lion cub activity is suggestive of positive emotional states, albeit infrequent and highly variable.

Grooming behaviour was not significantly different across the three facilities and yielded similar variability. Lions groom by licking and in affiliative behaviours, such as head rubbing (Matoba *et al.* 2013). Licking assists in reinforcing social bonds and has hygienic benefits (Schaller 1972), with head rubbing providing a tactile opportunity to show affection and communicate through scents (Bradshaw & Cameron-Beaumont 2000). Both forms of grooming between conspecific adults/subadults in captivity have been found to maintain and strengthen social bonds (Matoba *et al.* 2013), with cubs being the initiators of such social behaviour (Abell *et al.* 2013). The performance of allogrooming reflects a positive behaviour from a welfare perspective, and it was observed in the cubs. Autogrooming was the most dominant form of grooming by the cubs. It maintains health by keeping the individual clean but also aids thermoregulation, stimulates pheromones and decreases irritation and, as such, is vital for adaption and survival (Feusner *et al.* 2009). As with allogrooming, some autogrooming appears to reflect a positive emotional and behavioural state, but excessive autogrooming is indicative of a negative one. Human grooming, an obviously unnatural behaviour when compared to the wild lion, is however reflective of a bond existing between cub and keeper. Such reciprocal relationships have been known to exist between humans and farm animals and probably result in improved quality of life for both (Hemsworth 2003). There are reports of wild felids in captivity who rub and lick their human keepers, including those who, unlike lions, are solitary in behaviour (Cameron-Beaumont *et al.* 2002). This behaviour is strongly associated with human-reared felids (Mellen 1988). The reason why an animal should groom an unfamiliar human is yet to be determined. Perhaps it is a reciprocal behavioural reaction on the part of the animal who perceives the petting action as grooming or the animal soliciting favour, if it deems the human as a dominant presence. Human-associated grooming increased with human interaction frequency, which simply reflects more opportunity. Human interaction frequency did not have any effect on auto- or allogrooming.

Aggression was the least frequent behaviour category expressed by the cubs, with conspecific aggression occurring just a little more than human-directed aggression. McGlone (1986) suggests that aggression can be termed an abnormal behaviour when the form of aggression is not witnessed in the wild. But Dantzer and Mormede (1983) believe that aggressive behaviour can be considered abnormal when it results from a lack of control over the environment. Broom (1991) explains how fear is difficult to cope with and aggression may be an animals’ response. Aggression may also be displayed as a result of frustration when another behaviour is unable to be performed (Roper 1984). As such, a clear understanding of an aggressive response from a cub is required when evaluating its emotional state, be it towards conspecifics or humans. Cubs at Facilities pair AB exhibited increased flight behaviour as human interaction increased. Flight was also associated with human-directed aggression, thus linking the fight and flight responses. Both are viewed as responses to a state of fear (Boissy 1995). Animals need to have control over their environments through choice or manipulation, as it provides them with opportunities to avoid a negative stimulus. A lack of control results in fear, eliciting a flight, fight or undesirable behavioural response (McBride 1984). None of the lion cubs had access to a retreat space where they could retain control over their environments.

Non-nutritive sucking was the predominant form of abnormal behaviour at Facilities A and B. A behaviour typically observed in intensely farmed bovine calves, non-nutritive sucking is reduced when previous nutritive sucking bouts are for a sufficient time and when the non-nutritive sucking did not occur as a result of low feed levels, for example, when a meal was skipped (Rushen & de Passillé 1995). Milk source (cows’ milk or artificial milk) does not appear to influence non-nutritive sucking in calves (de Passillé *et al.* 1997). The desire to non-nutritively suckle was strongest for 10 min after having ingested milk. The satiating effect of the non-nutritive sucking was related to metabolic hormones (de Passillé & Rushen 1997). The survival of young depends on their successful suckling and de Passillé (2001) draws the link between this strong motivation and frustration if suckling is deprived, resulting in negative impacts on welfare. Given that the act of suckling is an appetitive behaviour, it should be provided for in captivity in order to alleviate stress (Carlstead & Shepherdson 2000). The performance of non-nutritive sucking in the lion cubs, indicates a desire to perform the behaviour.

Pacing is known to be the most dominant (97%) form of stereotype amongst carnivores (Clubb & Mason 2003) and was the most significant form of abnormal behaviour expressed by the lion cubs but was extremely variable in its possible causations. Environments which induce stereotypy reduce animal welfare. Mason (2006) suggests that stereotypy behaviour be classified as either: (i) a frustration-induced behaviour which is maladaptive, in that it is performed by a normal animal responding to an abnormal environment and can be reversed; or (ii) malfunction-induced behaviours which are associated with mental pathologies and impaired central nervous system functioning. Pacing, as a very common form of abnormal repetitive behaviour, was most evident at Facility C, when the cub cohort were split into adjoining enclosures and, as such, can be classified as a frustration-induced response to separation from conspecifics. This is supported by Bashaw *et al.* (2007) who state that carnivore pacing is associated with their inability to control sensory access to social partners.

Shaking and trembling, as observed by a cub at Facility B, represent a state of freeze, a defensive response not characterised by flight nor fight, as each are mutually exclusive (Eilam 2005). The freeze state reflects hypervigilance, associated with fear whilst a decision to then either flee or fight is being weighted up (Bracha 2004).

The fence-biting behaviour expressed by a cub at Facility C is an abnormal behaviour. Not all abnormal behaviours are harmful to the animal but do reveal a problem (Cooper & Mason 1998). This behaviour presented itself very infrequently and, should it have then developed further into a repetitive abnormal behaviour, would have been reflective of a state of ongoing frustration (Mason *et al.* 2007).

### Animal welfare implications

The welfare of animals used in tourism activities is widely understood to be compromised, and understanding the impact of such compromise is important if their welfare is to be addressed. An important aspect to consider is if workload contributes to such welfare compromise and to what extent this is the case. Human interaction frequency with petted lion cubs, in a tourism context, impacted upon several of the cubs’ behaviours causing, principally, a decrease in resting, sleeping and play and an increase in alert behaviour, grooming of humans and flight responses. Such behavioural responses did not reflect a coping response and indicate welfare compromise, even at a lower interaction frequency.
